# Isolation and characterization of extracellular vesicles from Broncho-alveolar lavage fluid: a review and comparison of different methods

**DOI:** 10.1186/s12931-019-1210-z

**Published:** 2019-10-30

**Authors:** Jonathan M. Carnino, Heedoo Lee, Yang Jin

**Affiliations:** Division of Pulmonary and Critical Care Medicine, Department of Medicine, Boston University Medical Campus, 72 E Concord St. R304, Boston, MA 02118 USA

**Keywords:** Extracellular vesicle (EV), Bronchoalveolar lavage fluid (BALF), Exosome, Microvesicle, Apoptotic body

## Abstract

Extracellular vesicles (EVs) are cell-derived membranous vesicles secreted by cells into the extracellular space, which play a role in cell to cell communication. EVs are categorized into 3 groups depending on their size, surface marker, and method of release from the host cell. Recently, EVs have become of interest in the study of multiple disease etiologies and are believed to be potential biomarkers for many diseases. Multiple different methods have been developed to isolate EVs from different samples such as cell culture medium, serum, blood, and urine. Once isolated, EVs can be characterized by technology such as nanotracking analysis, dynamic light scattering, and nanoscale flow cytometry. In this review, we summarize the current methods of EV isolation, provide details into the three methods of EV characterization, and provide insight into which isolation approaches are most suitable for EV isolation from bronchoalveolar lavage fluid (BALF).

## Introduction

### Extracellular vesicles

Extracellular vesicles (EVs) are membrane bound vesicles which play a role in cell to cell communication. EVs are released from host cells into extracellular space and have been found in many bodily fluids: urine, sputum, blood, saliva, breast milk, BALF, and more [1]. EVs contain and carry diverse materials such as lipids, proteins, RNA, glycolipids, and metabolites which originate from the host cells they are generated from [2, 3]. All categories of EVs have a lipid bilayer which encases the inner materials; this creates a stable internal environment and protects EVs from degradation by enzymes [4]. When EVs were first discovered, EVs were simply thought to be involved in the cellular excretion of byproducts, and were not given attention or studied very extensively [5]. Due to the similar characteristics of the major groups of EVs, the process of isolating and characterizing each type is difficult to do effectively [6]. Recently, it has become apparent that EV secretion, as well as EV-mediated pathways, are important in both normal biological processes and in several diseases processes [7]. Despite the increased interest and research into EV regulatory roles in disease pathology, the inconsistency in methodology for the collection, isolation, and analysis of EVs has posed a major barrier in further development of the field [8]. To combat this, the International Society for Extracellular Vesicles recently published a position statement offering guidelines to researchers in order to prevent variations across the studies of EVs [9].

### EV categories

**Fig. 1:**
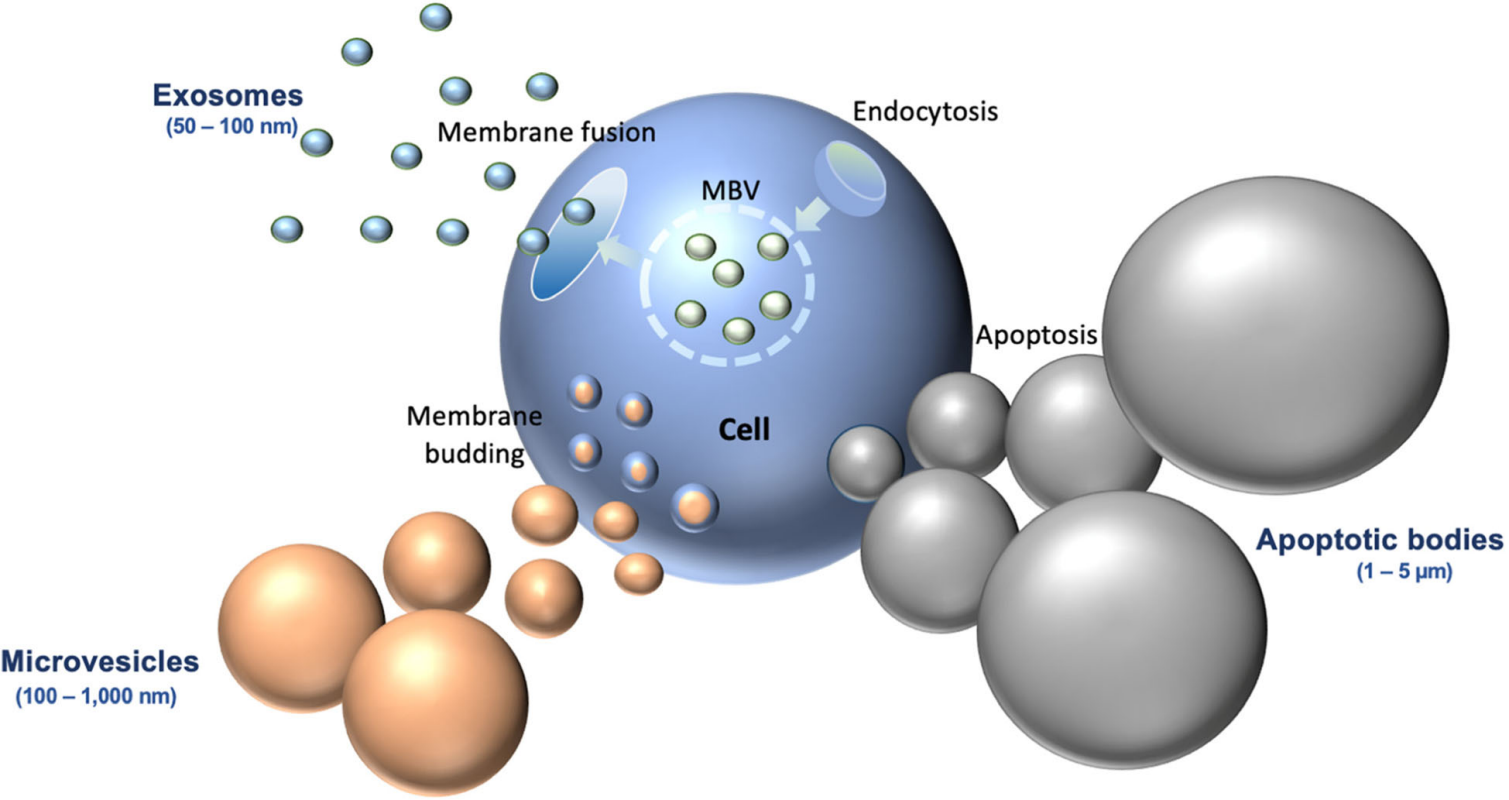
Schema of Each Major Category of EV. Schema highlighting the key difference in size and method of production between the three categories of EVs: Microvesicles, Exosomes, and Apoptotic Bodies. MBV: membrane-bound nanovesicles

Based on their mechanism of development, EVs are classified into three major groups: microvesicles, exosomes, or apoptotic bodies [10]. Figure 1**.** Microvesicles range in size from 100 to 1000 nm, and are formed from the outward budding of the plasma membrane of the host cell [11]. The membrane of microvesicles are known to contain larger amounts of cholesterol, diacylglycerol, and phosphatidylserine; and the main protein markers for this category of EVs are integrins, selectins, and CD40 [12]. Exosomes range in size from 30 to 150 nm, and are formed within the cell as multivesicular bodies, then eventually released into extracellular space after fusion with the cell membrane [11]. Exosome membranes are known to contain cholesterol, sphingomyelin, phosphatidylinositol, ceramide, and lipid rafts; and contain protein markers including CD63, CD9, CD81, and CD82, flotillin, TSG101, Alix, HSP60, HSP70, HSPA5, CCT2, and HSP90 [12]. Dying cells produce apoptotic bodies, which range from 50 to 5000 nm in size [13]. Apoptotic bodies contain exposed phosphatidylserine on their membranes, and their major protein markers include histones, TSP, and C3b [14]. A notable distinction between apoptotic bodies and the other two major EV groups is that apoptotic bodies also contain fragmented DNA and cell organelles from their host cell [15, 16].

### EVs as a potential biomarker

Immune cells, along with many other cell types, use EVs as a mode of cell to cell communication by transferring protein and genetic material, which exerts a regulatory role in the physiology and pathology of the cells in which they target [17]. This ability of EVs to transfer regulatory “messages” to other cells make them worthy of study as potential biomarkers [6]. MicroRNAs (miRNAs) have been extensively studied as they are known to play regulatory roles and serve as biomarkers in many diseases; therefore, the study of EV-containing miRNAs is understandably of specific interest [18, 19]. Development of bodily fluid-extracted biomarkers would be extremely beneficial as it would limit the need for collection of tissue samples and other invasive procedures [4]. Although, one disadvantage and barrier for now is that bodily fluids contain large amounts of soluble proteins and aggregates which pose contamination issues during EV isolation methods [7]. The isolation of highly pure EVs is essential to ensure the analysis of the results are not misleading due to contamination by viruses, lipoproteins, proteins, or other aggregates [18]. BALF, serum, and pleural fluid are all potentially good specimens which EVs can be isolated from to detect disease biomarkers in the future.

Emerging evidence displays that BALF EVs play an essential role in the pathogenesis of various lung diseases [20–35]. For example, BALF EVs have been reported to function as carriers of signaling mediator WNT5A, contributing to the pathogenesis of idiopathic pulmonary fibrosis [22]. Furthermore, BALF EVs generated by sarcoidosis patients have been reported to display pro-inflammatory effects [32]. Additional studies uncovering potential roles of EVs in many different disease processes can be found in Table 1.

**Table 1:** Partial Current Literature on BALF-EVs in Lung Diseases

Diseases/processes	Main conclusion	Author/Journal
Idiopathic Pulmonary Fibrosis (IPF)	Increased BALF-EVs function as carriers for WNT5A, and contribute to the pathogenesis of IPF	Martin-Medina et al.; AJRCCM 2018, Jul 25.
Asthma/COPD Bronchoconstriction	Mediate leukotriene conversion LTC4-LTD4	Lukic et al.; J Lipid Res 2016; 57:1659–69
Allergic Asthma	Leukotriene/cytokine production	Torregrosa Paredes et al.; Allergy. 2012 Jul;67(7):911–9
Allergy and vaccination	EVs can potentially induce tolerance	Prado et al.; J. Immunology 2008. 181
Asthma	EV-lipid profile as a biomarker	Hough et al.; Sci Report, 2018 10,340
COPD	EVs from PMN regulate the pathogenesis	Genschmer et al. Cell. 2019 Jan 10
Sarcoidosis	BALF EVs from sarcoidosis patients carry pro-inflammatory effects.	Qazi et al.; Thorax, 2010; 65
Lung transplant Acute rejection	The BALF EV profiles are altered in patients with acute rejection	Gregson et al.; AJRCCM 2015, Dec.15
Lung Cancer	BALF EVs contribute to lung cancer growth	Yang et al.: Frontier in Oncology 2019; April 12
Early stage Lung Ca	BALF EVs as a diagnostic marker	Kim et al.: Chest 2016 Oct Vol 150–4
Lung Cancer	Biomarker of cancer growth	Yang et al
ARDS	BALF-EV-miRNAs mediate inflammation and ALI	Sheller et al.: J Infectious Dis. 2019. Jan. 19th
Lung Injury	BALF-EV-miRNAs mediate sterile stimuli-associated ALI.	Lee et al.: J Immunology 2018
Pneumonia/sepsis	Macrophage-derived EVs regulate inflammation.	Soni et al. Thorax.2016 June 10
Pulmonary Hypertension	Exosomal 15-LO2 mediates hypoxia-mediated HTN	Zhang et al. Cell Death Dis. 2018 Oct 3;9(10):1022

In this review, we will cover a variety of EV isolation methods, and discuss the pros and cons of each method for isolating EVs from BALF and serum.

## Current methods to isolate EVs

### Differential centrifugation

Differential centrifugation is a conventional method which uses centrifugal force to separate contaminants from samples containing EVs. This separation technique involves separating and removing components other than EVs from a solution in a stepwise manner [36]. First, cell culture media or body fluids should be centrifuged at 300 g for 10 min at 4 degree Celsius to pellet dead cells and debris [5, 37]. The remaining supernatant is then centrifuged at 2000 g for 10 min at 4 degree Celsius to pellet ABs, and next, the remaining supernatant can be centrifuged at 10,000 g for 30 min at 4 degree Celsius to pellet MVs [37]. Lastly, the remaining supernatant is centrifuged once more at 100,000 g for 70 min at 4 degree Celsius to pellet Exos; the remaining pellet of ABs/MVs/Exos can be resuspended in PBS [5, 37]. The major advantages to this method are the low processing cost, the ability to work with large quantities of solution and isolate a large quantity of EVs at once, and the absence of additional chemicals needed for the technique [10, 38]. The need for ultracentrifugation equipment, the complexity of the stepwise technique, and that fact that efficiency of the technique is dependent on the type of rotor used are all disadvantages to differential centrifugation [10, 14]. Differential centrifugation can take between 140 and 600 min to complete [5, 38]. The sample volume parameters are dependent on the centrifuge rotary tubes used. Sample sizes can range from 1.5 mL to 25 mL depending on the availability of centrifuge and rotary tubes. Due to the ability to process large sample sizes at once, ultracentrifugation is likely a useful method for isolation of EVs from human samples. Additionally, for the isolation of EVs from BALF, ultracentrifugation has been proven to be a consistent method to isolate EVs from mouse BALF [39, 40].

### Density gradient centrifugation

Density gradient centrifugation isolates EVs into specific layers based on their buoyant density in solutions of either sucrose, iohexol, or iodixanol [41]. It is known that this method can successfully separate subcellular components such as peroxisomes, mitochondria, and endosomes into distinct layers within the density gradient solution [14]. Most density gradient protocols serve to further isolate EVs which have previously been partially isolated by centrifugation methods. One established protocol for density gradient centrifugation starts with loading 4 mL of Tris/sucrose/D_2_O solution to the bottom of a SW 28 tube, then carefully adding 25 mL of PBS containing partially isolated EVs to the top of this sucrose cushion, and subsequently centrifuging for 75 min at 100,000 g at 4 °C [42]. Next, 3.5 mL of the Tris/sucrose/ D_2_O cushion can be removed from the centrifuged tube and transferred to a new centrifuge tube [42]. This mixture can then be diluted with 60 mL of PBS, and centrifuged for 70 min at 100,000 g at 4 °C [42]. The resulting pellet contains the isolated EVs and should be resuspended in 50–100 μL of PBS [42]. The advantages to this method include: pure preparation, no contamination with viral particles, and absence of additional chemicals for the technique [14]. The disadvantages include: complexity, the need for ultracentrifugation equipment, and loss of sample during isolation [10, 14]. Density gradient centrifugation can be a time-consuming procedure, taking between 250 min to 2 days to complete [14, 42]. Similar to ultracentrifugation, sample size for density gradient centrifugation is mostly dependent on size of the centrifuge and rotary tubes available. This means sample volume parameters can potentially be within 1.5 mL and 25 mL, however, layering of gradients for this method may be difficult at low volumes and therefore a larger volume may be preferred. This is a suitable method for EV isolation from mouse BALF, however, due to the sample size may result in a lengthy processing time.

### Size-exclusion chromatography

Size exclusion chromatography makes use of porous beads to separate biomolecules based on their hydrodynamic radius [43]. This involves the filtration of a solution through a column of porous beads with radii smaller than the EV of interest [44]. During this process, fractions of solution will be eluted in order of decreasing size, and the fraction containing biomolecules with the size of the EVs of interest can be selectively isolated [14]. In one protocol, first 12 mL of Sepharose CL-2B is stacked into a 20 mL column, then rinsed and equilibrated with PBS [45]. Once the column is set up, 2 mL of cell culture media can be loaded into the column, and using PBS as an elution buffer, twenty 0.5 mL fractions should be collected from the column [45]. One clear issue with this technique is that there will likely be contamination of the sample by other molecules of similar size which elute at the same rate. The purity of preparation, preservation of vesicle integrity, and prevention of EV aggregation are notable advantages for using size-exclusion chromatography [45]. Also, due to the size overlap between categories, it is difficult to entirely isolate samples of EVs by their category. Additionally, this method allows for EVs to be isolated by their 3 respective categories based on their size differences. The disadvantages include: limitations on sample volume, the need for specialized equipment and a column, and complexity of the technique [45]. The processing time for size-exclusion chromatography is relatively much faster than most methods of EV isolation, taking 1 min per mL of solution [45]. It is recommended to use a sample volume of around 2–5% of the column volume, so sample volume is limited by the size of the column used for this protocol. This method is suitable for the rapid isolation of EVs from mouse BALF, however, if isolation of each category is desired, an alternative method should be utilized because of size crossover between ABs, MVs, and Exos.

### Commercial kits for polymer precipitation

Common commercial kits for EV isolation by Polyethylene glycol (PEG) precipitation are: ExoQuick (System Biosciences), Total Exosome Isolation Reagent (Invitrogen), ExoPrep (HansaBioMed), Exosome Purification Kit (Norgen Biotek), exoEasy (Qiagen), and miRCURY Exosome Isolation Kit (Exiqon) [14]. These kits all use solutions of superhydrophilic polymers, or PEGs, in order to decrease the solubility of EVs, forming a pellet precipitate. A pellet is formed by mixing the sample with a solution of PEGs, then centrifuging at low speed (about 1500 g) [44]. The pellet, consisting of EVs and some proteins contaminants, can then be resuspended in PBS and further analyzed. Commercial kits are relatively fast and have easy to follow protocols. Each kit is slightly different, however, most contain a PEG-based solution and utilize centrifugation as well. The advantages to this method are that it is a simple procedure and there is no need for additional equipment [18]. However, there are disadvantages as well, in that the kits are usually costly, may not be good for large samples of EVs, and there is a high concentration of impurities from isolation with these kits [44]. Another problem is that these kits cannot differentiate the three types of EVs, and therefore, during analysis we cannot identify which category of EV contained any packaged miRNA or protein cargo. Consequently, this method has a significant limitation if used to develop potential biomarkers, such as markers related to EV-cargo miRNAs. The run time for these commercial kits can be between 30 and 60 min or sometimes overnight depending on the kit used [14, 46]. Sample volume for these kits can range from 63 μL to 10 mL depending on the kit used and the type of sample processed. These kits are most commonly used for isolation of EVs from cell culture media, serum, or urine. These may be suitable for isolation from BALF as well, depending on the kit used and sample volume required. However, if isolation of EVs by category is required then alternative methods should be used.

### Precipitation with chemicals

Precipitation of EVs can be done with organic solvents, PEGs, sodium acetate, or protamine [47]. If using organic solvents such as acetone, chloroform, trichlo- roacetic acid, the ion-pairing effect can provide high efficiency when using these solutions to precipitate out EVs [48]. Precipitation by solutions of PEGs, as mentioned earlier, allows EVs and proteins to precipitate out of sample solution into a pellet, which can be further analyzed separately. This method tends to have many protein contaminants due to similar solubility. Using sodium acetate as a precipitation solution takes advantage of EVs negatively charged phosphatidylserine [49]. This method disrupts the hydration of EVs, leading to aggregation by the hydrophobic effect and forming a precipitate pellet [14]. A solution of protamine, a positively charged molecule, can be used to interact with and aggregate EVs because all EVs are known to be negatively charged [50]. After centrifugation, the mixture is gel filtered in order to remove the protamine and other impurities [14]. A common protocol for isolation of EVs by PEG precipitation is to combine cell culture media with PEG solution to create an 8% solution, followed by an ultracentrifugation wash at 100,000 g [51]. The resulting washed EV pellet can then be resuspended in sterile PBS [51]. Low cost, the simplicity of the procedure, and the ability to process samples of large volumes are all advantages to methods which use chemicals to precipitate out EVs [49, 51]. The overall disadvantages to these methods are the contamination issues with non-EV proteins, retention of chemicals or polymers, and the long processing time for some of these techniques [14]. Isolation with chemicals can be relatively quick depending on which solution is used (60–120 min), or overnight incubation may be necessary [18]. This method of EV isolation by precipitation with chemicals is able to be used on a wide range on sample volumes. For example, in EV isolation with PEG, it is only required that the final volume is 5–8% PEG. After incubation the mixture should then be centrifuged, therefore sample volume will be dependent on both size of sample and rotary tubes available, usually between 1.5 mL and 25 mL. For studies which don’t require isolation of EVs by category, this method is suitable for isolation from mouse BALF. However, if it is required to isolate each category of EV separately, alternative methods should be used.

### Immunoprecipitation

Immunoprecipitation can be used to take advantage of EV surface protein markers such as CD63, CD9, CD8 [6, 52]. In this method, sample solution is run through magnetic beads, which are coated with antibodies for common EV surface proteins [6]. This method allows for high selectivity, however, some types of EVs may elute with the solution and not be isolated if they do not contain the surface protein markers selected for. One protocol for EV isolation by immunoprecipitation involves running a resuspended EV pellet through a column containing beads coated in antibodies for CD63, CD9, and CD8 [52]. After this affinity-based isolation, the antibody beads are then washed to elute the EVs isolated [52]. This method is most commonly used for further isolation of EVs after a centrifugation method has been utilized. The overall advantages for this method seem to be the purity of isolated EVs and the high selectivity [53]. This method also allows for separation of different EVs based on their respective protein markers. The disadvantages are that selectivity may be too high, high cost, some difficulties with detachment of antibodies from EVs, and analysis of intact vesicles [6]. This method takes about 240 min to isolate EVs from a sample solution [6, 18]. Sample volume for EV isolation by immunoprecipitation is dependent on the amount of antibody coated beads used. For large sample volumes, high amounts of beads will be required, and vice versa for small sample sizes. Based on volume parameters, this method is also suitable for the isolation of EVs from mouse BALF. However, do to extremely high selectivity and possible issues in purity, this method may not be preferred. Additionally, it may require a large amount of antibody beads to process samples from mouse BALF.

### Ultrafiltration

Ultrafiltration uses porous membranes to trap molecules or particles of a specific size, allowing smaller molecules and particles to flow through the membranous filter [54]. This method is usually done in successive steps to isolate EVs of precisely the desired size [38]. Ultrafiltration is based on the particles size and mass, which means it is likely for proteins and other unwanted contaminants to be filtered with the desired EVs. One established protocol begins by concentrating 150 mL of cell culture media to 500 μL with a Centricon Plus-70 Centrifugal Filter (Ultracel-PL Membrane, 100 kDa) device using centrifugation at 3500 g at 4 °C [55]. Following this, the concentrate can be recovered with a reverse spin at 1000 g for 2 min [55]. The Centricon filter should then be washed with 30 mL of 70% ethanol by centrifugation at 3500 g, and then rinsed with 30 mL of PBS by centrifugation at 3500 g [55]. The simplicity of the procedure, the ability for concurrent processing of many samples, and the lack of limitations on sample volume are all notable advantages to this method [14, 54]. The disadvantages include: filter plugging which results in loss of sample, and sample contamination by proteins [38]. The technique of ultrafiltration for isolation of EVs usually takes about 130 min [38, 55]. This method does not have established limitations on sample volume, however, large sample sizes may lead to long processing times. Additionally, larger sample size will increase the likelihood of filter plugging, which will result in low yield. This method is suitable for the isolation of EVs from BALF, however, due to risks of low yield with higher sample volumes, this method may lead to difficulties in EV yield.

### Microfluidic technologies

**Fig. 2:**
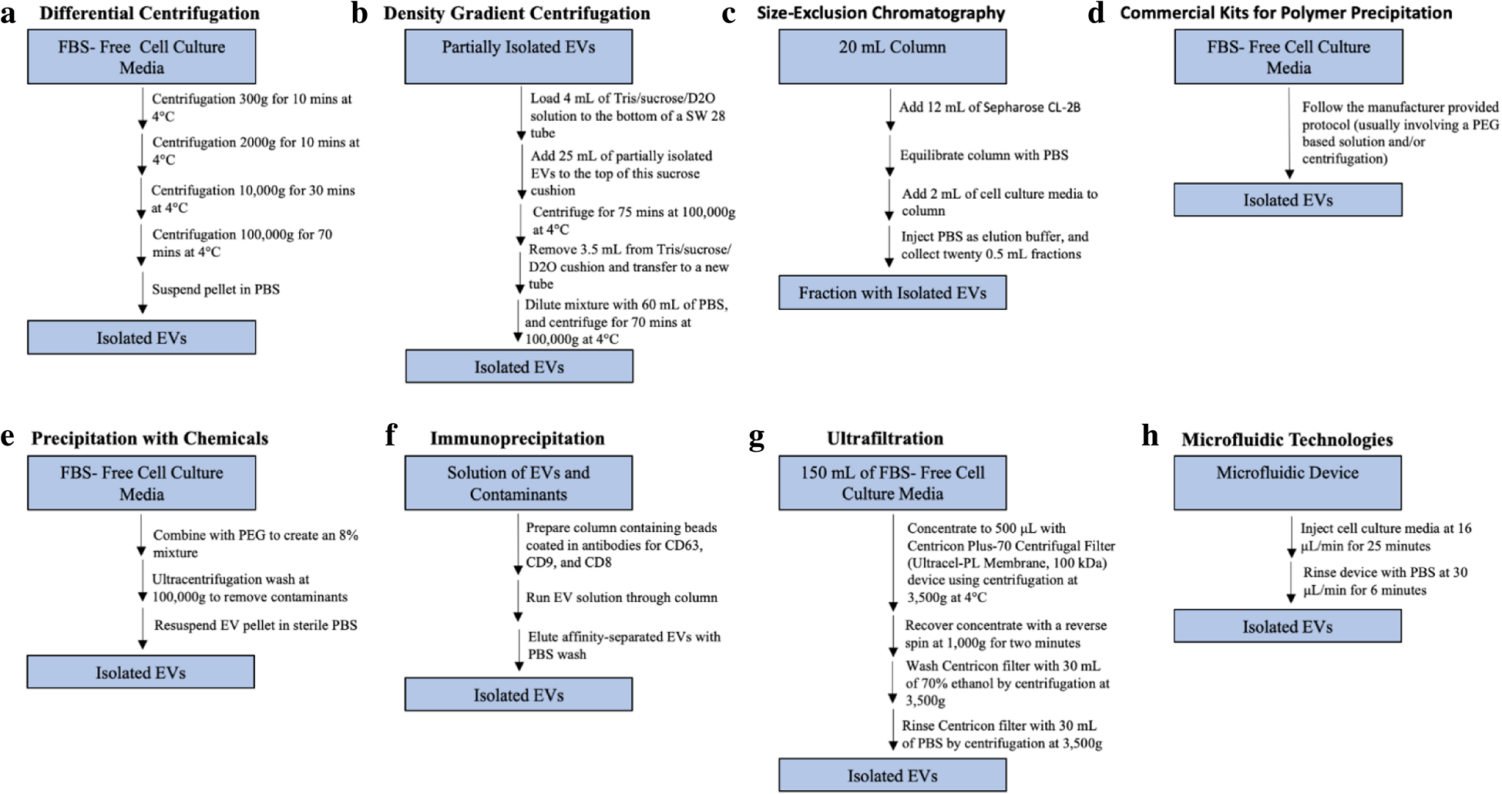
Flowchart of EV isolation methods. Summary of multiple different protocols for the isolation of EVs. **a**: Differential centrifugation, **b**: Density gradient centrifugation, **c**: Size-exclusion chromatography, **d**: Commercial kits for polymer precipitation, **e**: Precipitation with chemicals, **f**: Immunoprecipitation, **g**: Ultrafiltration, and **h**: Microfluidic technologies

Being relatively new technology, microfluidic devices direct the flow of liquids within small, micro-sized channels, which is able to separate and purify samples much more efficiently than any other sample separation method [42, 56]. These devices specifically capture and separate EVs by either immunoaffinity methods or by the entrapment within porous structures [57]. One microfluidic isolation protocol which can process samples up to 400 μL utilizes a microfluidic device with a straight flow channel of 19 mm in width, 20 μm in depth and 4.5 cm long with herringbone groves on its ceiling that are 50 μm wide and 10 μm deep [42]. For this protocol, cell culture media should be injected into the device at 16 μL/min for 25 min, then rinsed with PBS at 30 μL/min for 6 min [55]. EVs should adhere to the inner surface of the microfluidic device during initial injection, and be washed out by the following PBS injection. The resulting solution consists of the isolated EVs. Immunoaffinity methods involves the binding of particles by using antibodies which bind to surface proteins. The speed of processing with microfluidic technology is nearly instant. The advantages to this method include: rapidness of processing, sample purity, and processing efficiency [14, 18]. The high complexity of necessary devices, need for additional equipment, and high cost are all disadvantages to using microfluidic technologies [14]. The sample size required for this method is dependent on the length of the flow channel. Additionally, the rate of injection of sample size is low, and therefore for large sample sizes there will be a lengthy processing time. This method may be suitable for EV isolation from mouse BALF, however depending on the sample size, processing times may be lengthy. Due to the relatively large sample amount from mouse BALF, this method may end up becoming time consuming and complex. Figure 2 and Table 2.

**Table 2:** Techniques for EV isolation

Method	Processing time	Advantages	Disadvantages
Differential centrifugation	140–600 min	Cost Isolation from large volumes Absence of additional chemicals	Equipment (Ultracentrifugation) Complexity Efficiency is affected by the type of rotor
Density gradient ultracentrifugation	250 min–2 days	Pure preparations No contamination with viral particles Absence of additional chemicals	Complexity Equipment (Ultracentrifugation) Loss of sample
Size-exclusive chromatography	1 ml/min + column	Pure preparations Preserves vesicle integrity Prevents EV aggregation	Limitations on sample volume Specialized equipment and column Complexity
Commercial kits for polymer precipitation	30–60 min or overnight	Simple procedure No need additional equipment	Cost (especially for diluted samples, such as urine) Impurities
Precipitation with chemicals (polymers, polyethylene glycol, protamine, sodium acetate)	60–120 min or overnight	Cost Simple procedure Possibility of processing samples with large volume	Contamination with non-EV proteins Retention of chemical or polymer Long duration (sometimes)
Immuno-precipitation (CD9, CD63, CD81 or specific cell type marker)	240 min	Purity and high selectivity	High selectivity Cost Difficulties with detachment of antibodies Analysis of intact vesicles
Ultrafiltration (nanomembrane or filters with a pore diameter of 0.8–0.1 μm)	130 min	Simple procedure Allowing for concurrent processing of many samples No limitations on sample volume	Filter plugging (loss sample) Contamination (proteins)
Microfluidic technologies		Rapidness Purity Efficiency	Complexity of devices Additional equipment Cost

### EV isolation from BALF

This review focuses specifically on the isolation of EVs from bronchoalveolar lavage fluid (BALF). The main issue for BALF is the limited amount of specimen. Therefore, some techniques which require large volume cannot be used to isolate EVs from BAL.

Isolation and identification of BALF-derived EVs is still at the very initial, or “concept” stage, and protocols are not yet well established. According to the International Society of Extracellular Vesicles (ISEV), and many other published papers, three main subgroups of EVs (ABs, MVs, and Exos) can be enriched by 2000–3000 g (AB), 10,000–16,000 g (MVs), and 100,000–120,000 g (Exos) force of sequential centrifugation. We have shown that the MV population is the main type of BALF EV and falls into the size range of 100–400 nm using sequential centrifugation [39, 40]. On the other hand, Exos are 50–150 nm sized BALF EVs [39, 40]. Notably, in our previous study [58], we found that the EV protein markers were differentially expressed among the ABs, MVs, and Exos. Especially TSG101, which is a critical protein for generating MVBs, was highly expressed in the Exosome population. On the other hand, caveolin-1, which is a central component in lipid-raft microdomains, was predominantly expressed in MV population, suggesting that the BALF EV isolation using sequential centrifugation technique is a reliable and convincing method.

During the processes of BALF EV isolation using UC and PEG precipitation, there are several basic advances which should be reported. To begin with, it would be ideal if the EV purification is performed immediately after the BALFs are obtained. We monitored the critical EV aggregation and size modification when the EVs were purified from frozen BALF samples, and it is very hard to recuperate their unique original character. Secondly, we suggest delicate sonication of the purified EVs utilizing a water-bath sonicator before EV analyses are conducted. It significantly helps to disperse the EV aggregates, which are possibly generated during the sequential centrifugation or EV freezing/thawing step, and get accurate and consistent results. Finally, long-term storage of the isolated EVs is not recommended. We found that remarkable destruction or loss of EV components, including proteins and RNAs, occurs during the long-term storage of the EVs.

## Characterization of EVs

Isolated samples of EVs also often contain a mixture of contaminants consisting of small organelles, lipids, cholesterol, and other undesired microparticles [58]. It is essential to verify the purity of isolated EV samples in order to validate the accuracy of the experimental results derived from processing of the samples. It is possible that contamination of isolated EVs may lead to abnormal or misleading data, therefore, checking the sample purity is a crucial step in properly analyzing EVs.

Additionally, characterizing the category of EV (Exo, MV, or AB) may be important for the analysis and interpretation of results from EVs. A reason for this is because some compositions (RNA or protein) may exist more in certain categories of EVs than others. For example, it has been previously reported that serum exosomes contain a very small amount of miRNAs per Exo, and therefore are unlikely to possess a biological purpose [59]. Exosomes are much smaller (30–150 nm) and formed by endosomal origin, whereas MVs are much larger (100–1000 nm) and formed by the outward budding of the lipid membrane [11, 60]. Due to this distinction in quantity of contents, MVs may play a greater role in communicating cell injury and could be a more valuable prospect for future studies.

Another important characteristic of EVs which should be analyzed is the integrity of the isolated microparticle. In order for EVs to have a future potential use particularly in the development of drug delivery, it is critical that EVs maintain their integrity and efficacy after multiple cycles of being frozen and thawed in order to have the ability to be developed into a pharmaceutical product for the future [61]. Isolated EVs from different cells, which preserve both their integrity and effectiveness after many freeze-thaw cycles, are good candidates to be used for drug delivery in the future.

Additionally, EVs can be characterized to determine the cell type from which the EV originated from based on detection of EV surface antigens that are identical to the surface antigens found on its cell of origin [58]. This information is useful for study as we can then determine based on the cell type of origin which tissue type the EV originated from, and therefore which organ is under stress. By backtracking the EV to their site of origin, in the future we can further examine and understand the etiology of diseases, specifically the role of EVs in communicating stress leading to systemic inflammation spreading to organs around the body.

Moreover, characterization of EVs also allows us to determine the number of EVs released by count [61]. A specific total count of EVs released by cells under stress lets us determine if there is an induction of EVs released to communicate the injury to nearby cells or tissues. This data, along with information about the contents within each EV (RNAs or proteins) may provide further insight into the role EVs play in the communication of cell damage.

### Dynamic light scattering (DLS)

DLS measures size of particles based on their Brownian motion in solution; the basis of Brownian motion is that lighter particles will diffuse faster, and that speed is relative to particle size. This method is used specifically to measure size distribution of EVs and their zeta potential as well [7]. This technique illuminates particles using a laser; the light scattering by the particles and intensity changes are detected, then further analyzed to determine particle size and distribution within solution [62]. Dynamic light scattering can measure particles smaller than 10 nm or larger than a micron, and provides an intensity-based distribution of EVs. DLS provides an average value of relatively uniformly sized particles, and therefore would not be the best technique for a heterogeneous solution of EVs [18]. DLS is able to measure the diameter range of analyzed EVs (1 nm-6 μm), but provides no biochemical data or report about the cell from which the EV originated [63]. Notably, DLS is also much less accurate for heterogenous mixtures of EVs and provides the most precise data when testing isolated samples of Exos, MVs, or ABs [64].

### Nanoparticle tracking analysis (NTA)

Similar to DLS, NTA measures EV concentration and size distribution on the basis of Brownian motion as described before [65]. In NTA, a laser beam is directed into solution, and the Stokes-Einstein equation is used to measure the mean velocity of the particles, which can then be used to calculate the size of the particles [4]. One major issue with this method is that NTA cannot distinguish an EV from a different particle, meaning any particle that displays similar Brownian motion to EVs will be included in analysis using NTA [18]. Notable features of NTA is that the particle-by-particle measurement can provide a number-based distribution, NTA can give the percentage of EVs by number of particles, and NTA often offers a higher resolution than other characterization techniques. Overall, NTA can be used to characterize the size, count, and distribution of EVs ranging from 1 to 1000 nm [66]. Of note, NTA does have reported difficulty in characterization of heterogenous samples of EVs, and is most suitable for samples of isolated Exos and MVs [67]. NTA is unable to detect and characterize isolated samples of ABs due to its particle size constraint.

### Nanoscale flow Cytometry (nanoFACS)

Flow cytometric analyses of bead-bound EVs allows for the analysis of specific EV populations of interest using antibodies that precisely recognize EVs from heterogeneous samples. However, this method cannot evaluate the complex profiles of subsets of EVs with multiple labels assessed for each EV. Therefore, a high-resolution flow cytometry method for analyzing and sorting individual EVs and other nanoscale particles (e.g. liposomal products, HIV) is required to improve the single EV analysis. NanoFACS combines measurements from high sensitivity multiparametric scattered light and fluorescence to analyze and sort EVs individually [58, 68]. One of the obvious advantages is that nanoFACS can separate and distinguish the nano-sized particles from instrument noise and background. Similar to both DLS and NTA, nanoFACS is able to provide data on the size, count, and distribution of EVs provided in the sample used [68]. Moreover, this method can also use specific fluorescently labeled antibodies to stain EV surface proteins, and therefore determine the cell type the EV originated from [58, 68]. This notable tool can be extremely valuable for the study of activation markers on both Exos and MVs. With this useful information, researchers can gain insight about EV populations originating from a particular cell type, which may be involved in different disease etiologies. Another noteworthy feature of nanoFACS, which distinguishes it from standard flow cytometry, is its ability to differentiate actual EVs from other nanoparticles, contaminants, or artifacts which may have become part of the sample during processing [58]. This feature provides an accurate display of data representing only the EVs characterized. Table 3.

**Table 3:** Comparison of EV Characterizing and Analyzing Techniques

Technique	DLS	NTA	nanoFACS
Feature	Techniques based on the Brownian motion of and light scattering from the particle		Techniques based on the dual forward scatter and single forward scatter
	Measure the diffusion coefficient and derive the size from that diffusion coefficient. So both are influenced by shape in the same manner		Enhanced Forward Scatter (eFSC) technology
	Ensemble measurement provides an intensity-based distribution	Particle-by-particle measurement provides a number-based distribution	Expression of certain EV surface epitopes which can be stained by specific fluorescently labeled antibodies
	Can provide the % by intensity of particles	Can give the % by number of particles	Valuable tool to study cell-type specific surface proteins or activation markers on exosomes.
	To measure particles smaller than 10 nm or larger than micron	Can often provide higher resolution,	To gain insights about EV populations originating from a particular cell type

### Transmission Electron microscopy (TEM)

TEM is a form of microscopy which uses beams of electrons to produce a magnified image of a specimen or sample. Compared to standard light microscopes, transmission electron microscopes produce images with significantly higher resolution. TEM is a useful tool for characterizing the morphology, size, and phenotype of EVs [69]. This method can also be used to check the purity of sample by providing a high resolution image to distinguish EVs from similarly-sized non-EV particles that may also reside in the sample after EV isolation [70]. Lastly, use of TEM is a critical step in the characterization of EVs because it provides visual verification that the sample used for experimentation is actually EVs. This confirmation is important in the interpretation of data and refutes any possibility that contaminants may have skewed experimental results as well.

## Conclusions

EVs are a relatively new area of research and there is still much work to be done in order to develop a more thorough understanding of their role in communicating cell stress. Analysis of BALF is a common method of studying pulmonary diseases and etiology. By creating a gold standard method for EV isolation from BALF, and the development of new EV characterization methods in the near future, hopefully we can advance our understanding of the role EVs play in the diseases processes of many pulmonary illnesses.

## Data Availability

Data sharing is not applicable to this article as no datasets were generated or analyzed during the current study.
